# Seasonal and Year-Round Distributions of *Bactrocera dorsalis* (Hendel) and Its Risk to Temperate Fruits under Climate Change

**DOI:** 10.3390/insects13060550

**Published:** 2022-06-16

**Authors:** Zhaoke Dong, Yitong He, Yonglin Ren, Guanjin Wang, Dong Chu

**Affiliations:** 1 Engineering Research Center for Precision Pest Management for Fruits and Vegetables of Qingdao, Key Lab of Integrated Crop Pest Management of Shandong Province, College of Plant Health and Medicine, Qingdao Agricultural University, Qingdao 266109, China; zhaoke_dong@126.com (Z.D.); hoyt.he@murdoch.edu.au (Y.H.); 2 College of Science, Health, Engineering and Education, Murdoch University, 90 South St., Murdoch, WA 6150, Australia; y.ren@murdoch.edu.au; 3 Discipline of Information Technology, Murdoch University, Perth, WA 6150, Australia; guanjin.wang@murdoch.edu.au

**Keywords:** *Bactrocera dorsalis*, invasive pests, potential distribution, risk assessment, early prediction

## Abstract

**Simple Summary:**

The oriental fruit fly *Bactrocera dorsalis* (Hendel) is a pest species in the Tephritidae family that damages many fruits and vegetables. Dispersal of *B. dorsalis* is mediated by human activities (e.g., trade) and climate change, and it can cause serious damage to crops in newly invaded regions. Previous studies mainly focused on the areas potentially suitable for year-round reproduction, but it is unclear where the seasonal and year-round suitable areas are in the world. We used ecological niche models to predict the potential seasonal and year-round distribution areas of *B. dorsalis*. Bioclimate factors contributed differently to these two kinds of distributions. In the future, the areas suitable for *B. dorsalis* will increase, and the range will likely expand northward from existing locations. The spread of *B. dorsalis* in the seasonally suitable areas could threaten the production of some temperate fruits, including apples, peaches, pears, and oranges.

**Abstract:**

*Bactrocera dorsalis* (Hendel) is an important pest to fruits and vegetables. It can damage more than 300 plant species. The distribution of *B. dorsalis* has been expanding owing to international trade and other human activities. *B. dorsalis* occurrence is strongly related to suitable overwintering conditions and distribution areas, but it is unclear where these seasonal and year-round suitable areas are. We used maximum entropy (MaxEnt) to predict the potential seasonal and year-round distribution areas of *B. dorsalis*. We also projected suitable habitat areas in 2040 and 2060 under global warming scenarios, such as SSP126 and SSP585. These models achieved AUC values of 0.860 and 0.956 for the seasonal and year-round scenarios, respectively, indicating their good prediction capabilities. The precipitation of the wettest month (Bio13) and the mean diurnal temperature range (Bio2) contributed 83.9% to the seasonal distribution prediction model. Bio2 and the minimum temperature of the coldest month (Bio6) provided important information related to the year-round distribution prediction. In future scenarios, the suitable area of *B. dorsalis* will increase and the range will expand northward. Four important temperate fruits, namely, apples, peaches, pears, and oranges, will be seriously threatened. The information from this study provides a useful reference for implementing improved population management strategies for *B. dorsalis*.

## 1. Introduction

Biological invasions threaten food safety and biodiversity, and such threats are expected to increase with increased international trade and shifts in suitable habitats associated with climate change [1,2]. Billions of dollars in economic losses have resulted from agricultural pest invasions worldwide [3,4]. Fruits are often attacked by tephritid fruit flies, which are invasive worldwide and can cause severe losses [5]. Infested fruits are not suitable for sale, consequently reducing marketability. The oriental fruit fly *Bactrocera dorsalis* (Hendel) is a serious pest species in the family Tephritidae. This species is highly polyphagous and attacks many species of fruits and vegetables, such as orange, apple, pumpkin, and cucumber species [6]. It overwinters in the soil as pupae and its survival rate is related to winter temperature. *B. dorsalis* typically occurs in tropical and subtropical areas [7,8]. In mainland China, it is distributed in provinces located south of the Yangtze River [9]. Defining the geographic distribution of pest species and their cryptic variation is important for understanding and managing their movement and dispersal potential [10]. 

Detection and eradication of *B. dorsalis* is necessary, and often needs to be repeated, e.g., the situation in California [11]. To prevent their establishment, information about a species’ likelihood of invasion is necessary. Ecological niche models (ENMs) have often been used to predict the potential distributions of species [12]. When modeling the habitat suitability of *B. dorsalis* population, researchers usually refer to population survival in terms of year-round occurrence, and neglect the seasonal occurrence [10]. However, seasonal populations of *B. dorsalis*, without control measures, during favorable seasons can also cause substantial losses to fruits; for example, *B. dorsalis* damaged peaches in autumn 2019 but did not survive in winter in Heze city, Shandong Province, China (field observations). Therefore, when using ENMs to predict their potential distribution, it is necessary to consider the seasonal distribution alongside year-round distribution.

Climate change has caused substantial modifications in the geographical distribution of many species [13]. The Sixth Assessment Report (AR6) produced by the Intergovernmental Panel on Climate Change (IPCC) states that global warming of 1.5 °C and 2 °C will be exceeded during the 21st century unless major reductions in CO_2_ and other greenhouse gas emissions occur in the coming decades [14]. Under this climate forecast, the potential damage risk to temperate fruits caused by *B. dorsalis* has not currently been assessed.

The purpose of this study was to evaluate (1) the potential distribution of *B. dorsalis*, both in terms of seasonal and year-round occurrence, and (2) the risk areas of seasonal occurrence of *B. dorsalis* on temperate fruits. This information will be valuable for determining how to manage the invasion and spread of *B. dorsalis* to suitable regions.

## 2. Materials and Methods

### 2.1. Databases Used in Modeling

#### 2.1.1. Species Occurrence Data of *B. dorsalis*

Distribution records of *B. dorsalis* were obtained from the literature [7,8,9,10], as well as from online databases such as the Global Biodiversity Information Facility (GBIF, http://www.gbif.org/, accessed on 1 June 2021) and CAB International (https://www.cabi.org/isc/datasheet/17685, accessed on 1 June 2021). We eliminated duplicates and reduced the effects of spatial autocorrelation by rarefying records with a distance of 50 km, which meant the nearest two record sites were more than 50 km [15,16,17,18]. A total of 397 unique occurrence records were retained. Spatial thinning was conducted using SDMtoolbox 2.3 for ArcGIS [19]. Occurrence records in Northern China represent occurrences during favorable seasons, and do not indicate persistence throughout the year. Therefore, we divided the occurrence data into seasonal and year-round occurrence. We excluded seasonal occurrence sites when modeling suitable year-round habitats.

#### 2.1.2. Environmental Data

Current bioclimatic variables were obtained from the WorldClim database at a resolution of 10 arcmin (http://www.worldclim.org, accessed on 1 June 2021). The WorldClim dataset is derived from measurements of monthly climate data collected from weather stations across the world between 1950 and 2000 [20]. These climate data were used to gain a stable description of the sites. The predictor variables used to assess current climate conditions were selected among 19 bioclimatic variables (see the supplementary file for details of these variables). These variables capture annual climatic ranges and limiting factors that are known to influence species geographic distributions. Selecting important predictor variables is an essential step for model fitting. Two sets of variables (set 1: Bio1, Bio2, Bio5, Bio6, Bio12, Bio13, and Bio14; set 2: Bio1, Bio2, Bio5, Bio6, Bio12, and Bio15) were selected following the procedure suggested by [21]. These variables are mainly used to describe temperature variation and precipitation variation. The first set of bioclimatic variables was selected based on a previous study that successfully modeled the distribution of other *Bactrocera* species [21]. The second set of bioclimatic variables was a modified version of set 1, created by replacing the variables Bio13 and Bio14 with Bio15. Since we did not know which variable set would be the best predictor for *B. dorsalis,* we tried these two sets of variables to gain a reliable result.

We used the model data developed by the Coupled Model Intercomparison Project (CNRM-CM6-1) of two Shared Socio-economic Pathways (SSPs: 126 and 585) for future climate scenarios. According to the Intergovernmental Panel on Climate Change (IPCC), there are four Shared Socio-economic Pathways (SSPs: 126, 245, 370, and 585) which comprise high-priority scenarios for the Sixth Assessment report by the IPCC [14]. These data refer to the low greenhouse gas emission scenarios (SSP126) and the high emission scenario (SSP585). We downloaded these data at a resolution of 10 arcminutes from https://www.worldclim.org/, accessed on 1 June 2021.

### 2.2. Protocol of MaxEnt Modeling

#### 2.2.1. Background Selection

MaxEnt uses pseudo-absence data drawn randomly from a geographically defined background instead of actual absence records to define environmental conditions where the species has not been recorded. The background from which pseudo-absences are drawn can, however, significantly influence the model results [22]. Due to this, it is recommended that the background be restricted to the region in which the species would reasonably be expected to occur [23].

**Figure 1:**
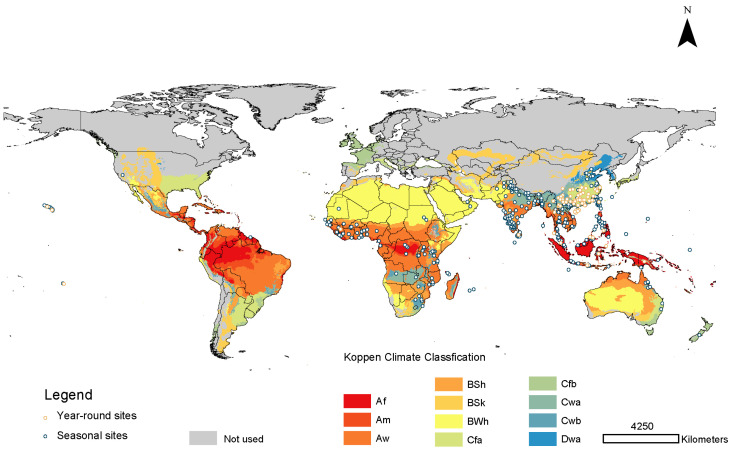
Seasonal and year-round occurrence points of populations of *Bactrocera dorsalis* used in the modeling process. Colors refer to Köppen–Geiger classifications, while the grey areas represent areas not used in models. The letter codes for climate classes are as follows. Af: equatorial rainforest, fully humid; Am: equatorial monsoon; Aw: equatorial savannah with dry winter; BSh: hot semi-arid climate; BSk: cold semi-arid climate; BWh: hot desert climate; Cfa: humid subtropical climate; Cfb: temperate oceanic climate; Cwa: monsoon; Cwb: subtropical highland climate or temperate oceanic climate with dry winters; Dwa: monsoon-influenced, hot summer, humid continental climate. Details of the calculation of the Köppen–Geiger climate classification are described in [25].

For broadly distributed invasive species (where dispersal measures are largely unknown), it may be best to select backgrounds based on bioclimatic zones representing little inhibition to the accessible area beyond broad climate types [22]. We selected the background study area by intersecting the occurrence localities with Köppen climatic zones downloaded from CliMond (http://www.climond.org, accessed on 2 June 2021) at the spatial resolution of 10 arc-minutes [24,25]. Climate zones containing one or more distribution records were used to restrict the background during model training (Figure 1). Ten thousand pseudo-absences were then drawn from an area defined by Köppen–Geiger polygons, within which one or more distribution records were located.

#### 2.2.2. Parameter Set and Model Evaluation

MaxEnt has two main modifiable parameters: (1) feature classes and (2) regularization multiplier. Feature classes can be used to build very complex and highly nonlinear response curves [26]. A feature is a function of an environmental variable and, in MaxEnt, it can be a combination of six classes: linear (L), quadratic (Q), product (P), hinge (H), and threshold (T). As parsimonious models can be generated using different combinations of feature classes, five of these combinations were tested in this study: L, H, LQ, LQH, and LQHPT. The regularization multiplier is a parameter that adds new constraints [27]. It has been demonstrated that less complex and transferable models can be built by tuning the regularization multiplier to values higher than the default of MaxEnt [28,29]. Therefore, in addition to the MaxEnt default setting, regularization multiplier values of 3 and 5 were also tested in the development of the models. We combined regularization multipliers and feature classes to assess a total of 186 models for two environmental datasets.

The performance of the models was assessed using partial receiver operating characteristic (ROC), omission rates, and AICc [30]. Partial ROC was calculated instead of the full area under the ROC curve because the latter is not appropriate in ecological niche modeling [31], and partial ROC represents a more suitable indicator of statistical significance [30]. Best models were selected according to the following criteria: (1) significant models with (2) omission rates ≤ 5%. Then, models with delta AICc values of ≤2 were chosen from among this model set as the final models.

#### 2.2.3. Model Projection to Predict the Potential Distribution

Once the parameter combination yielding the best model was determined, the MaxEnt model was run with all the known occurrences from native and invaded areas and projected onto the remaining parts of the world to predict the potential distribution of *B. dorsalis*. The final model was run for 10 replications and the output provided in a logistic format to increase the accuracy and reliability of modeling results. After using the current climatic data to model the spatial extent of suitable habitat for *B. dorsalis*, modeling projections were performed for future climate scenarios to predict the extent of suitable habitats in the future (2040 and 2060). 

The final model was run with the logistic output and maps were built using the 10% training presence threshold (TP10). Areas above the TP10 were referred to as suitable, and below were unsuitable. The potential distribution ranges of suitable habitats were divided into three levels between the above threshold, and one with Jenks natural breaks, corresponding to low, medium, and high suitable habitats. We used the tool “reclassify” in ArcGIS (v.10, ESRI 2011) to create the figure of the analysis result.

### 2.3. Spatial Analyses for Quantifying the Area at Risk of Damage

The worldwide acreages of orange *Citrus sinensis*, apple *Malus domestica*, pear *Pyrus communis*, and peach *Prunus persica* were obtained from [31]. To quantify the production areas at risk of attack, the suitability map of *B. dorsalis* was intersected against maps of fruit acreage across the world. Areas that overlapped with the predicted distributions of *B. dorsalis* were considered to be at risk of attack.

## 3. Results

### 3.1. Model Evaluation

A total of 186 models were tested for each type of distributions. For the seasonal distribution, 146 statistically significant models met the omission rate criterion, of which two also met the AICc criterion at the same time. For the year-round distribution, three significant models met the omission rate criterion, of which one also met the AICc criterion at the same time. The best model had the lowest AICc values and omission rates (Table 1). We selected the models in which set 1 participated as the best models for seasonal and year-round occurrence, respectively. All of these models included seven variables (set 1: Bio1, Bio2, Bio5, Bio6, Bio12, Bio13, Bio14). However, their feature class and regularization multipliers differed. The mean AUC training values for both models were 0.860 and 0.956, respectively, indicating that both models performed well.

The most important factors limiting the seasonal distribution of *B. dorsalis* were the precipitation of the wettest month (Bio13, 56.7% variation) and the mean diurnal temperature range (Bio2, 27.2% variation). The cumulative contributions of these factors were as high as 83.9% (Table 2). In contrast to the seasonal model, the most important factors limiting the year-round distribution were the mean diurnal temperature range (Bio2, 57.7% variation), the minimum temperature of the coldest month (Bio6, 19.4% variation), and the mean annual temperature (Bio1, 8% variation) (Table 2).

The thresholds of suitable habitat (predicted probability, TP10) were above 0.288 for seasonal distribution and 0.2548 for year-round distribution. The response curve shows how each environmental variable affects the prediction (Figures S1 and S2). For seasonal distribution, Bio13 higher than 143.56 mm, and Bio2 lower than 12.19 °C were suitable. For year-round distribution, Bio2 lower than 11.04 °C and a Bio6 ranging from −11.48 to 15.28 °C were suitable.

### 3.2. Current Potential Distributions

**Figure 2:**
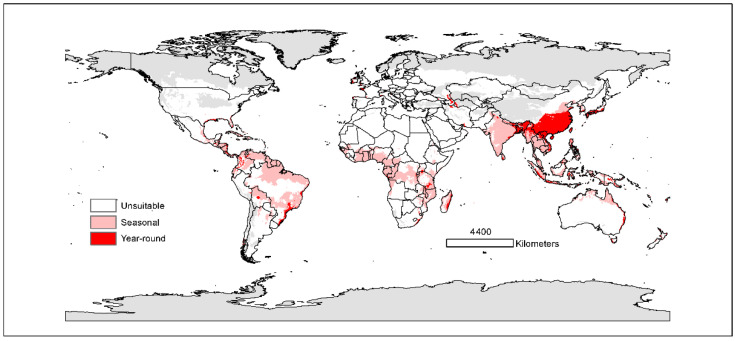
Predicted current distribution model of *B. dorsalis* for the seasonal distribution (pink) and year-round distribution (red). White represents unsuitable habitat area; grey represents areas not used for prediction.

The seasonal distribution model for *B. dorsalis* showed that suitable areas covered parts of Southeast Asia, Australia, Central America, South America, and Africa (Figure 2). Habitats that are currently highly suitable for seasonal distribution were concentrated in Southern China, Southeast Asia, and coastal areas of other continents. For year-round distribution, highly suitable areas were mainly in Southern China, Eastern India, and a small part of Central Asia. Some coastal areas of Brittany and the British Isles were also predicted to be suitable, although only at a low level (Figures S3 and S4).

With the current environmental variables, the total area of potentially suitable habitat for seasonal distribution was estimated to be 28.6 × 10^6^ km^2^. Among these areas, about 5.11 × 10^6^ km^2^ (about 17.87% of the total suitable area) exhibited high habitat suitability (Table 3). The potentially suitable year-round habitat area was 7.10 × 10^6^ km^2^, which accounted for only 24.84% of the seasonal distribution.

### 3.3. Future Changes in Suitable Areas

Future climate changes will be generally favorable for *B. dorsalis* (Table 3), and additional suitable areas will be available under two climate change scenarios (SSP126 and SSP585). The potential range will primarily expand north from the currently occupied areas. Under the SSP126 climate scenario, MaxEnt predicted that *B. dorsalis* would have increased areas of suitable habitats by 2040 and 2060 (Figures S3 and S4). In addition, the Mediterranean would become suitable for *B. dorsalis* in the future. Under the SSP585 climate scenario, MaxEnt predicted that *B. dorsalis* gains suitable habitat areas in North America, Africa, and the Mediterranean by 2040 and 2060 (Figure S3). The suitability of habitats in Asia would also increase. Overall, we saw an increasing pattern in suitable habitats compared with current habitats (Table 3).

### 3.4. Risks to Global Fruit Production

**Figure 3:**
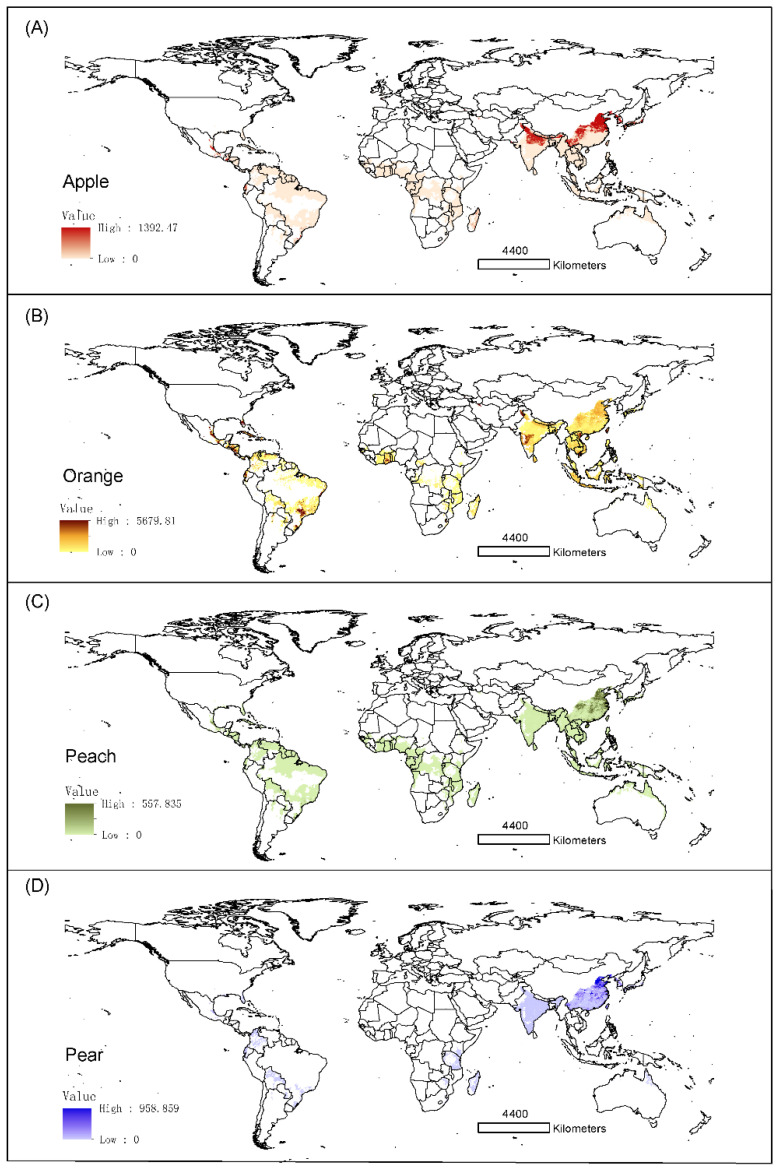
Areas of four fruit species at risk of attack by *B. dorsalis* in the potential seasonal distribution habitats. The value in each plot shows the harvested area (hectare). (**A**) apple; (**B**) orange; (**C**) peach; (**D**) pear.

**Figure 4:**
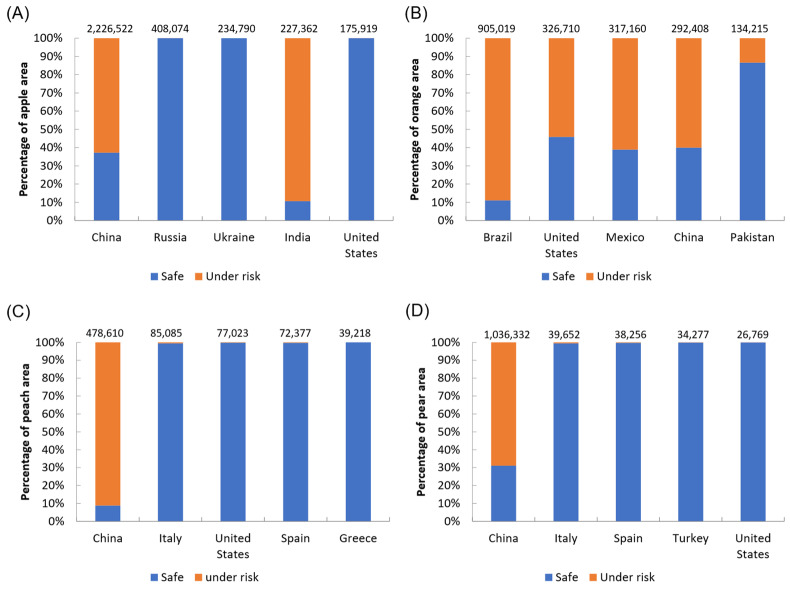
Based on the current potential seasonal distribution, percentage of safe and risk areas in terms of attack by *B. dorsalis* for four important fruit species: (**A**) apple; (**B**) orange; (**C**) peach; (**D**) pear. The number on each bar refers to the harvested area (hectare). Countries in each plot are ordered according to the harvested area, from largest to smallest.

The four temperate fruits (apple, peach, pear, and orange) are at risk of *B. dorsalis.* The production of orange is the one that is at higher risk, with almost 78% of its production area within the suitable range of *B. dorsalis*, followed by apple (51%), pear (38%), and peach (30%). Brazil, the United States, Mexico, and China had more than 50% of orange area at risk. China also had the largest areas at risk in terms of the apple, peach, and pear production (Figure 3 and Figure 4).

**Figure 5:**
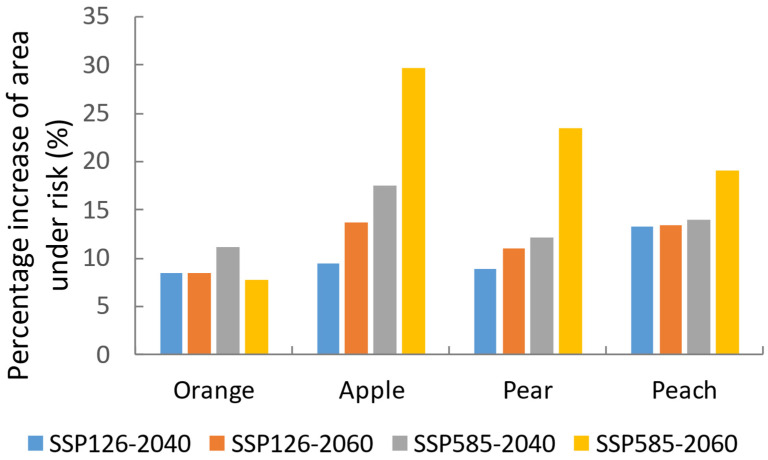
Percentage increase in area at risk of attack by *B. dorsalis* for four temperate fruit species, comparing four future climate scenarios/years to the current situation. Under the current situation, risk area for orange is 1.96 million hectares, apple 1.75 million hectares, pear 0.78 million hectares, and peach 0.52 million hectares.

Under SSP126–2040, SSP126-2060, SSP585-2040, and SSP585-2060 climate scenarios, the areas in which all these fruits would be at risk of attack were predicted to increase (Figure 5).

## 4. Discussion

In this study, we performed a detailed analysis of the seasonal and year-round suitability of areas for *B. dorsalis* under different climate scenarios. The results obtained could be an important step in formulating a feasible strategy for pest management. Our models show that *B. dorsalis* has a wide range of potential seasonal distribution, including Southern and Southeastern Asia, Central and South America, and parts of Africa, as well as coastal Australia and New Zealand. In China, parts of Hebei, Henan, and Shandong provinces are also predicted to be suitable for seasonal distribution. In contrast, the potential year-round distribution was mainly in Southern and Southeastern Asia, South America, and Australia. The seasonal distribution of *B. dorsalis* usually refers to places that are suitable only in the warm season because the coldest month and length of day impact survival. During the warm season, temperate fruit (e.g., apple) is ripe, so the risk of damage to these fruits should be noted. 

Distinguishing the seasonal and year-round distributions of *B. dorsalis* is crucial for its management. For example, in seasonal distribution areas, control strategies can focus on preventing the invasion of this pest through trade or transport; for instance, the detection and eradication efforts in California show that the prevention work may need to be performed constantly [11]. Monitoring efforts should emphasize the corridors or routes connecting the current overwintering areas to each other or to all suitable habitats. On the other hand, in year-round distribution areas, control strategies need to focus on reducing overwintering populations. When invasions occur in some seasonal distribution areas, molecular tools can be used to trace their dispersal pathways and determine which year-round distribution areas are the sources of invasive populations [9]. Further research should focus on a regional scale to figure out the accurate borders of year-round distribution and develop strategies to prevent the dispersal of *B. dorsalis* from year-round distribution areas to seasonal areas.

*B. dorsalis* is endemic and widespread in tropical areas of Southeast Asia [6,32]. The risk of infestation by this species on temperate fruits may be overlooked when it is considered only as a tropical pest. We believe that the seasonal distribution of this pest may also cause damage. The four temperate fruits analyzed (apples, peaches, pears, and oranges) are at risk of being attacked by *B. dorsalis* outside tropical areas. The adults of *B. dorsalis* have been reported to be mobile (about 50 km travelling distance), and their larvae spread mainly through human activities (e.g., fruit trade) [8]. Therefore, this pest is highly likely to move from year-round to seasonal areas. Detection and eradication programs need to be prepared in suitable seasonal areas.

Previous models showed the high percentage of climatically suitable areas in South America and Africa [32,33,34]. Compared with these previous studies, our results predicted a lower percentage of suitable habitats in South America and Africa. In addition, our seasonal model shows more northern distributions in China, Europe, Korea, and Japan. According to previous studies [32,33,34], there is also a current risk of *B. dorsalis* establishment in the coastal regions of the Mediterranean. In contrast, our model shows low suitability for *B. dorsalis* along the Mediterranean coast. However, our results show that parts of the coastal areas in Western Europe are predicted to be suitable for *B. dorsalis* (Figure 2). It is hard to attribute the differences to certain factors. Different modeling methods may generate different results. Our predicted year-round distribution matched that of previous studies, but covered slightly smaller areas. It may be due to our limited year-round occurrence data. Some occurrence records in Africa and South America were not included in our year-round modeling because of the lack of verification of their year-round status. Future work should focus on the details of seasonal and year-round distribution patterns of *B. dorsalis*.

The spread of *B. dorsalis* has motivated the implementation of risk assessments and control measures [35]. In China, *B. dorsalis* began spreading northward in the early 21st century, and this expansion continues [34]. *B. dorsalis* is now moving into central areas of China that were previously considered climatically unsuitable because of cold winter conditions [36]. Distribution records from Central and Northern China were not included in the model validation process [6]; it has been hypothesized that these records may represent transient populations. The MaxEnt model used by [34] included records from central provinces (Jiangsu, Anhui, and Hubei). Our model also included some locations in Northern China (Beijing, Hebei, and Shandong), where *B. dorsalis* is occasionally reported (GBIF, https://www.gbif.org/, accessed on 1 June 2021). In Central China, a small proportion of overwintering pupae may result in a small number of adults early in the season and ensure population survival. This survival may constitute selection pressure for increased cold tolerance of *B. dorsalis* and future range expansion [37]. The northern boundary of the overwintering sites in China (Jiangsu, Anhui, and Hubei) would benefit from increased monitoring.

Finally, we found that the precipitation of the wettest month and mean diurnal temperature range were important factors for predicting seasonal distribution. In contrast, the minimum temperature of the coldest month and mean diurnal temperature range were most useful for predicting year-round distribution (Table 2). It is not surprising that different bioclimatic predictors contribute to predicting seasonal and year-round distributions. However, the mean diurnal temperature range provided useful information for both distribution predictions. This predictor uses recorded temperature fluctuations within a month to capture the diurnal temperature range, and it can provide information related to the relevance of temperature fluctuations [38]. 

## 5. Conclusions

Our model predicted a significantly high risk of *B. dorsalis* seasonal infestations to apples, peaches, and pears in China, and oranges in Brazil, the United States, and China. The spread of *B. dorsalis* in seasonally suitable areas could result in significant economic losses to producers, since more than a half of the planting areas are within these areas (Figure 4). China accounts for almost 54.6% of global apple production, and 62.77% of its planted area is within the potential seasonal distribution of *B. dorsalis*. In India, 89.39% of apple planting areas are at risk of *B. dorsalis* infestation. Future climate changes will generally be favorable for *B. dorsalis*, and additional suitable areas will be available. We also found the percentage of risk area for the four studied fruits would increase in the future (Figure 5). Preventing invasions is more cost-effective than eradicating or controlling the invading species once it has become established in a region [39]. Our results show areas where *B. dorsalis* is most likely to invade. Future research should be focused on developing priority areas for interception or guidance for the installation of detection traps.

## Figures and Tables

**Table 1:** Summary of performance statistics of best models for predicting seasonal and year-round distributions of *Bactrocera dorsalis*. The relative regularization multiplier (RM), feature classes (FC), sets of predictors (Pred. Sets), and AICc values are shown. Delta AICc and weight AICc of models with default settings are relative to the selected models.

	RM	FC	Pred. Sets	Partial ROC	Omission Rate	AICc	Delta AICc	Weight AICc
**Seasonal**	3	PH	Set 1	0	0.04	7422.17	0.00	0.51
	1	LQP	Set 2	0	0.04	7423.00	0.82	0.34
**Year-round**	1	LQP	Set 1	0	0.03	1385.06	0.00	0.99

**Table 2:** Percentage contributions and permutation importance of the bioclimatic variables included in the MaxEnt models for seasonal occurrence and year-round occurrence of *Bactrocera dorsalis*. Here we list the results from best models based on set 1.

		Seasonal		Year-Round	
Variable Name	Variable Description	Contribution (%)	Permutation Importance	Contribution (%)	Permutation Importance
Bio1	Annual mean temperature	5.3	13.7	8.0	20.5
Bio2	Mean diurnal temperature range	27.2	32.3	57.7	26.9
Bio5	Max temperature of warmest month	2.6	0.8	5.0	11
Bio6	Min temperature of coldest month	1.6	8.4	19.4	30.9
Bio12	Annual precipitation	1.3	7.3	2.7	7.3
Bio13	Precipitation of wettest month	56.2	36.2	5.0	2
Bio14	Precipitation of driest month	5.9	1.3	2.2	1.4
Bio15	Precipitation seasonality	-	-	-	-

**Table 3:** Portions of different classes of potential distribution area for seasonal and year-round occurrence under current and four future climate scenarios/years (×10^6^ km^2^).

	Seasonal				Year-Round			
	Unsuitable	Low	Medium	High	Unsuitable	Low	Medium	High
Current	248.74	11.83	11.66	5.11	270.35	3.21	1.91	1.86
SSP126-2040	242.24	12.58	13.83	8.69	268.46	4.01	2.77	2.10
SSP126-2060	241.08	12.81	14.26	9.19	268.32	4.12	3.07	1.83
SSP585-2040	240.13	13.16	14.43	9.63	267.96	4.20	2.57	2.60
SSP585-2060	236.79	13.30	15.03	12.21	268.07	4.13	2.72	2.41

SSPs are the Shared Socio-economic Pathways that provide a range of distinct end-of-century climate change outcomes; the years refer to the monthly values averaged over 20-year periods (2021–2040, 2041–2060).

## Data Availability

The data presented in this study are available in supplementary materials.

## Supplementary Materials

The following supporting information can be downloaded at: https://www.mdpi.com/article/10.3390/insects13060550/s1, Supplementary material 1: Figure S1: Response curves showing the relationship of MaxEnt predicted probability of seasonal occurrence of *Bactrocera dorsalis* to environmental variables. Figure S2: Response curves showing the relationship of MaxEnt predicted probability of year-round occurrence of *Bactrocera dorsalis* to environmental variables. Figure S3: Predicted seasonal distribution of *B. dorsalis* under two climate scenarios (SSP126 and SSP585) and two future years (2040 and 2060). Figure S4: Predicted year-round distribution of *B. dorsalis* under two climate scenarios (SSP126 and SSP585) and two future years (2040 and 2060).
