# Supplementary material for: Seasonal and Year-Round Distributions of Bactrocera dorsalis (Hendel) and Its Risk to Temperate Fruits under Climate Change
Source: Insects. 2022 Jun 16;13(6):550. doi: 10.3390/insects13060550 (PMC9225012; doi:10.3390/insects13060550)
Supplement: Supplementary file 1 [file insects-13-00550-s001.zip › insects-1632977-supplementary.pdf]

insects

MDPI

Supplementary Materials

Seasonal and Year-Round Distributions of *Bactrocera dorsalis* (Hendel) and Its Risk to Temperate Fruits under Climate Change

Zhaoke Dong [1], Yitong He [1,2], Yonglin Ren [2], Guanjin Wang [3] and Dong Chu [1,*]

[1] Engineering Research Center for Precision Pest Management for Fruits and Vegetables of Qingdao, Key Lab of Integrated Crop Pest Management of Shandong Province, College of Plant Health and Medicine, Qingdao Agricultural University, Qingdao 266109, China; zhaoke_dong@126.com (Z.D.); hoyt.he@murdoch.edu.au (Y.H.)
[2] College of Science, Health, Engineering and Education, Murdoch University, 90 South St., Murdoch, WA 6150, Australia; y.ren@murdoch.edu.au
[3] Discipline of Information Technology, Murdoch University, Perth, WA 6150, Australia; guanjin.wang@murdoch.edu.au
* Correspondence: chinachudong@qau.edu.cn (D.C.)

Citation: Dong, Z.; He, Y.; Ren, Y.; Wang, G.; Chu, D. Seasonal and Year-Round Distributions of *Bactrocera dorsalis* (Hendel) and Its Risk to Temperate Fruits under Climate Change. *Insects* **2022**, *13*, 550. https://doi.org/10.3390/insects13060550

Academic Editor: Grzegorz A. Buczkowski

Received: 31 March 2022
Accepted: 14 June 2022
Published: 16 June 2022

Publisher's Note: MDPI stays neutral with regard to jurisdictional claims in published maps and institutional affiliations.

Copyright: © 2022 by the authors. Licensee MDPI, Basel, Switzerland. This article is an open access article distributed under the terms and conditions of the Creative Commons Attribution (CC BY) license (http://creativecommons.org/licenses/by/4.0/).

Table S1. The 19 bioclimatic variables and their descriptions, see https://www.world-clim.org/data/bioclim.html for details.

Variables	Descriptions	Unit
Bio1	Annual Mean Temperature	°C
Bio2	Mean Diurnal Range	°C
Bio3	Isothermality. Mean diurnal temperature range / annual temperature range *100	-
Bio4	Temperature Seasonality	coefficient of variation
Bio5	Max Temperature of Warmest Month	°C
Bio6	Min Temperature of Coldest Month	°C
Bio7	Temperature Annual Range	°C
Bio8	Mean Temperature of Wettest Quarter	°C
Bio9	Mean Temperature of Driest Quarter	°C
Bio10	Mean Temperature of Warmest Quarter	°C
Bio11	Mean Temperature of Coldest Quarter	°C
Bio12	Annual Precipitation	mm
Bio13	Precipitation of Wettest Month	mm
Bio14	Precipitation of Driest Month	mm
Bio15	Precipitation Seasonality	coefficient of variation
Bio16	Precipitation of Wettest Quarter	mm
Bio17	Precipitation of Driest Quarter	mm
Bio18	Precipitation of Warmest Quarter	mm
Bio19	Precipitation of Coldest Quarter	mm

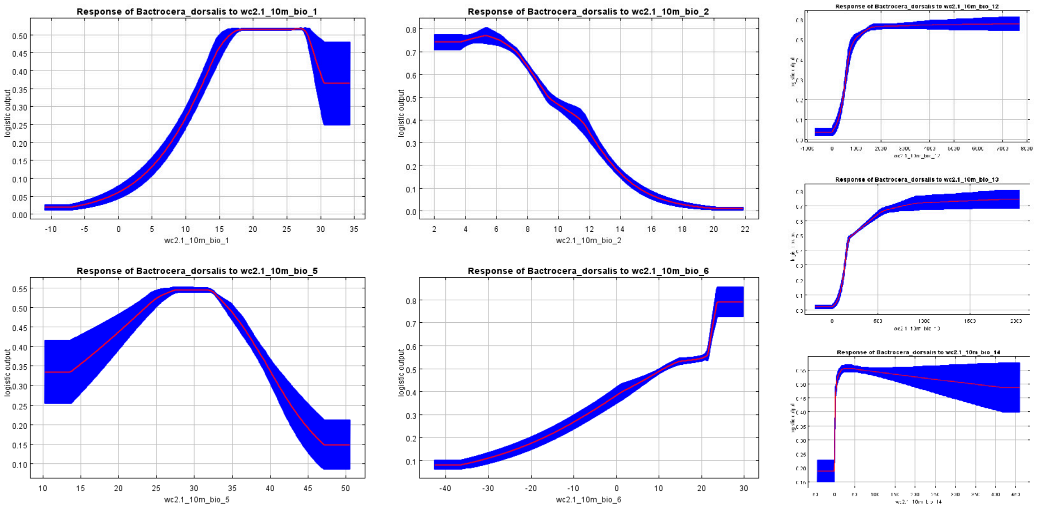

Figure S1. Response curves showing the relationship of MaxEnt predicted probability of seasonal occurrence of Bactrocera dorsalis to environmental variables. The values shown on the y-axis are the predicted probability of suitable conditions. The values on the x-axis refer to the corresponding bioclimate variable. Each curve is made by generating a MaxEnt model using only the corresponding predictor variable, disregarding all other variables.

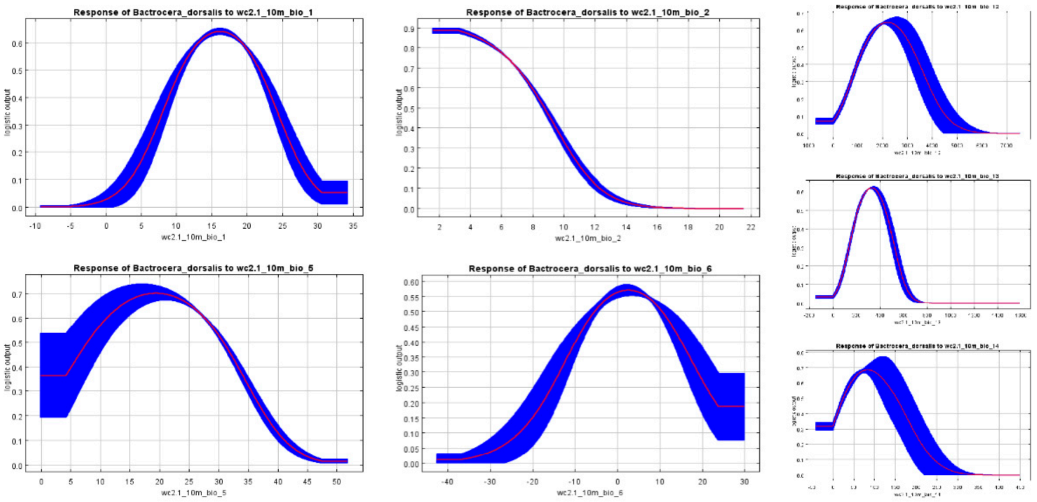

Figure S2. Response curves showing the relationship of MaxEnt predicted probability of year-round occurrence of Bactrocera dorsalis to environmental variables. The values shown on the y-axis are the predicted probability of suitable conditions. The values on the x-axis refer to the corresponding bioclimate variable. Each curve is made by generating a MaxEnt model using only the corresponding predictor variable, disregarding all other variables.

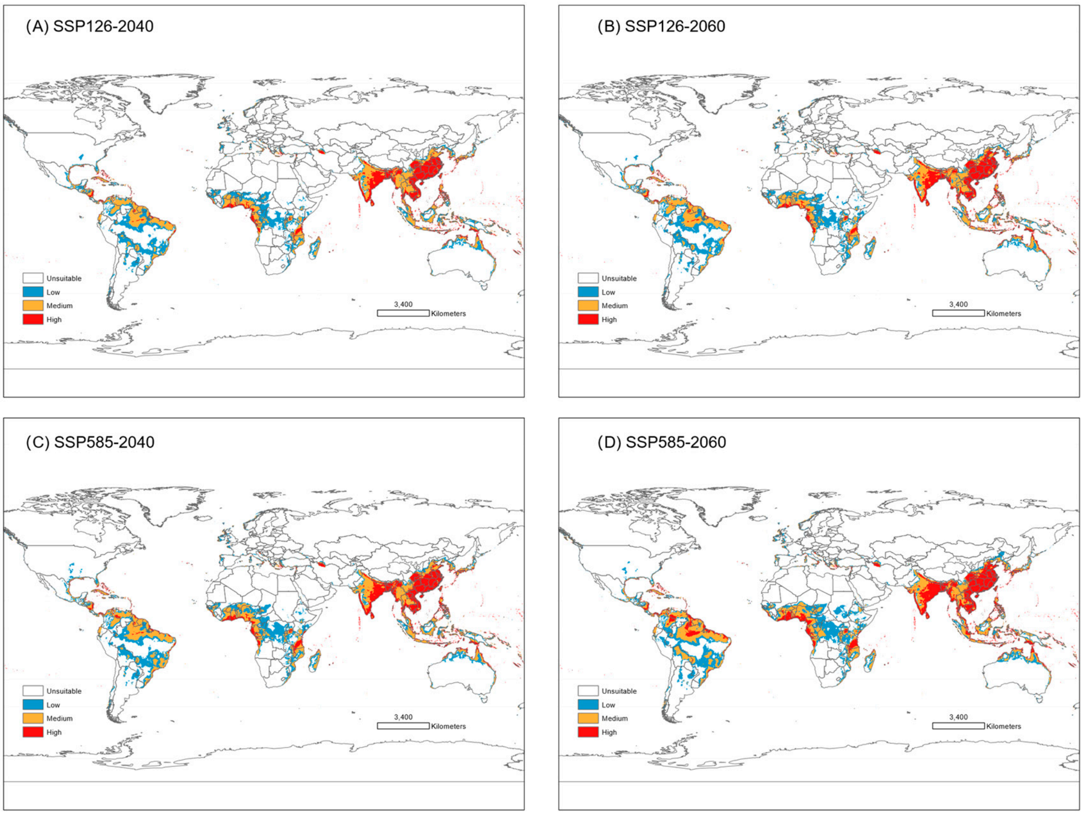

Figure S3. Predicted seasonal distribution of *B. dorsalis* under two climate scenarios (SSP126 and SSP585) and two future years (2040 and 2060). White, unsuitable habitat area; blue, low suitability area; orange, medium suitability; red, high suitability. (A) SSP126 to 2040, (B) SSP126 to 2060, (C) SSP585 to 2040 and (D) SSP585 to 2060.

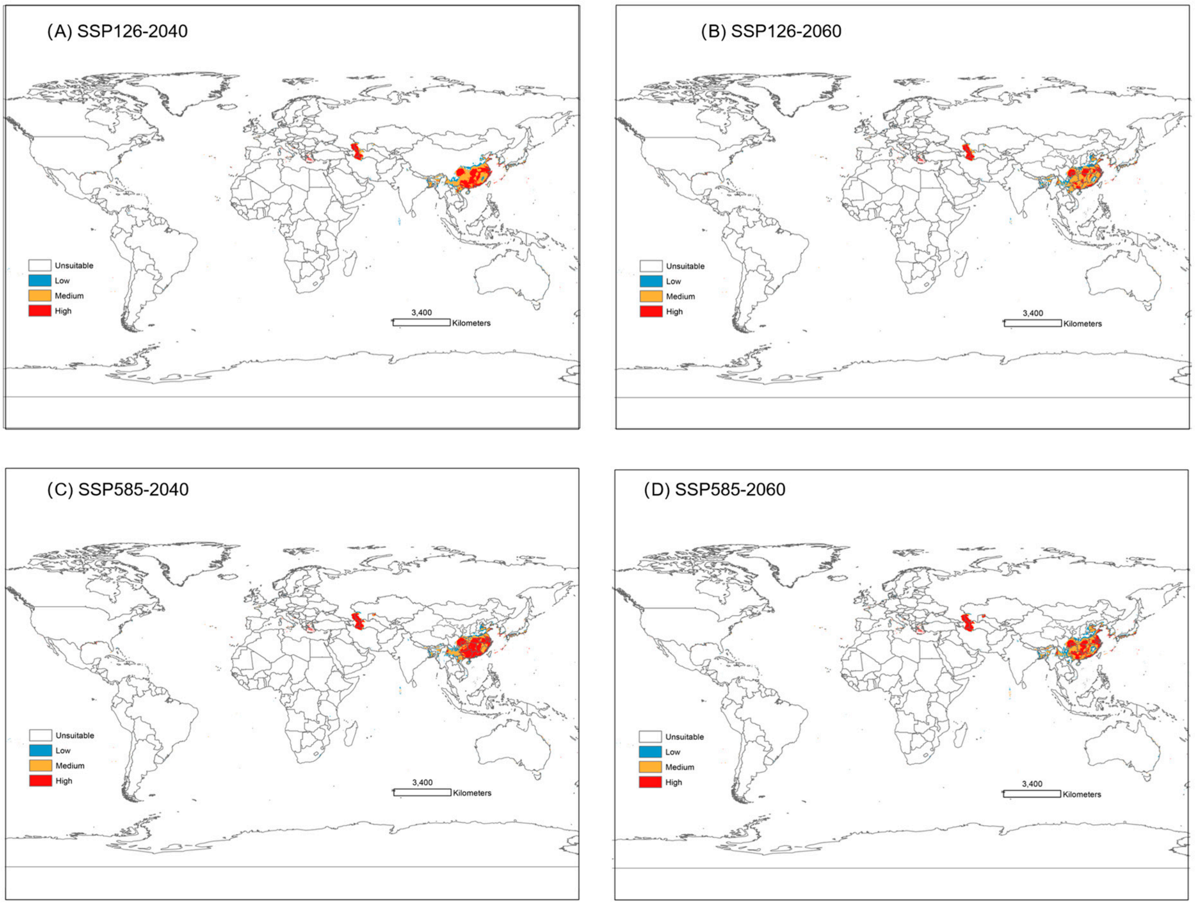

Figure S4. Predicted year-round distribution of *B. dorsalis* under two climate scenarios (SSP126 and SSP585) and two future years (2040 and 2060). White, unsuitable habitat area; blue, low suitability area; orange, medium suitability; red, high suitability. (A) SSP126 to 2040, (B) SSP126 to 2060, (C) SSP585 to 2040 and (D) SSP585 to 2060.
